# Postpartum Myasthenic Crisis in a Patient with SLE: A Case Report and Literature Review

**DOI:** 10.31138/mjr.33.4.449

**Published:** 2022-12-31

**Authors:** Keerthivardhan Yerram, Ritasman Baisya, Phani Kumar Devarasetti

**Affiliations:** Clinical Immunology and Rheumatology, Nizams Institute of Medical Sciences (NIMS), Hyderabad, India

**Keywords:** myasthenia gravis, systemic lupus erythematosus, anti-AChR antibodies, pyridostigmine

## Abstract

**Background:**

Systemic lupus erythematosus (SLE) is a multisystem autoimmune disease characterized by the presence of numerous autoantibodies while Myasthenia Gravis (MG) is an organ-specific autoimmune disease. The coexistence of both diseases is rarely reported in the literature.

**Case presentation:**

We report a case of a 29-year-old female SLE patient with chief manifestations of nephritis, inflammatory polyarthritis and cytopenia presented with postpartum shortness of breath and dysphagia requiring emergency intubation and difficulty in weaning. Later she developed chronic respiratory acidosis with bilateral ptosis. Her diagnosis of myasthenia was confirmed with a positive neostigmine test and nAChR antibodies. She was given 5 cycles of PLEX and pyridostigmine with significant improvement of symptoms and extubated safely.

**Conclusion:**

It is one of the rare case reports of SLE preceding MG with significant improvement by anticholinergic therapy.

## INTRODUCTION

Systemic lupus erythematosus (SLE), a prototype multisystem autoimmune disease, is characterised by the presence of numerous auto-antibodies resulting in chronic inflammation. Myasthenia gravis (MG), an organ specific autoimmune disease, is characterized by the presence of specific autoantibodies against the presynaptic membrane receptors like Nicotinic acetylcholine receptor (nAChR), muscle-specific tyrosine kinase (MuSK) or lipoprotein receptor-related protein 4 (LRP4).^1^ In 13–22 % of the cases MG is associated with other autoimmune diseases including Rheumatoid arthritis, SLE, Hashimoto thyroiditis, Graves’ disease, and pernicious anaemia. This association is more common in female and patients with early onset disease.^2^ The prevalence of MG in the SLE population is estimated at 1–2%.^3^ SLE and MG share common features including higher prevalence among young women, positive antinuclear antibodies, and relapsing and remitting course.^1,4,5^ Although both MG and SLE are relatively common autoimmune disorders, their coexistence is rarely reported. Here we have reported our experience of a SLE patient with postpartum myasthenia crisis.

## CASE HISTORY

A 29-year-old female patient with a 2-years history of SLE with inflammatory polyarthritis, nephritis (ISN-RPS class 3), leukopenia, mood disturbance (depression) presented in the emergency department with shortness of breath and dysphagia two days after her first delivery. SLE was clinically quiescent during pregnancy, and she delivered a male baby at term without complications. Shortness of breath was sudden in onset and rapidly progressive without orthopnoea, chest pain, cough, oliguria, or oedema. She had significant dysphagia to both solids and liquids. There were no other signs of SLE disease activity. She had one episode of focal seizure with secondary generalisation and was intubated for airway protection. On examination she was hypertensive, tachypnoeic with laboured breathing with 92 % saturation at room air. Laboratory testing revealed normal CPK (80), low complement with normal anti-dsDNA antibodies (previously reported positive), anti-cardiolipin antibody (aCL-IgG/IgM) negative. On the third day, despite a normal chest radiograph with clinical improvement, extubation was deferred owing to significant respiratory acidosis (pH 7.28, pCO2 60) in arterial blood gas analysis. Within the next few days, the patient developed acute onset pupillary sparing bilateral ptosis (left > right) with left lateral rectus palsy. Acute myasthenic crisis was suspected involving extra-ocular and respiratory muscles. Her quantitative myasthenia gravis score was 14. Atropine neostigmine test showed significant improvement in ptosis. Serology showed positive AChR antibodies (4.6, cut off 0.5) and diagnosis of myasthenia gravis was confirmed. Treatment was started with pyridostigmine and plasma exchange (PLEX). Progressively she was weaned off from the ventilator, extubated, and shifted to an oxygen mask. There were no further desaturation episodes. After the 5^th^ cycle of PLEX, she improved significantly and was discharged in stable condition.

## DISCUSSION

The coexistence of SLE and MG is rare but not coincidental. The American College of Rheumatology Research Committee in 1999 described the 19 NP syndromes (12 CNS and 7 PNS) appearing in SLE; MG is one of the seven peripheral manifestations of NPSLE.^6^ It is estimated that while almost half of SLE patients are affected by NPSLE, the prevalence of MG as peripheral NPSLE is less than 1–2%.^3^ Similarly, the prevalence of SLE is higher in MG patients in the general population and is estimated to be up to 8%.^1,7,8^ In most patients (62%), MG preceded SLE diagnosis.^1^

Very few cases are published in literature in which SLE diagnosis was concomitant or preceded MG.^1^ A comprehensive literature review of case reports and case series in which SLE was associated with MG was performed. Literature searches were conducted in PubMed. Keywords and mesh searches included the following: SLE, MG, myasthenia crisis, acetylcholine receptor antibodies.

In a case series of five cases by Bekirkan Kurt et al.^9^ mean age at SLE and MG onset were 33.2 & 41 years respectively. Four cases had nephritis, two had haematological and one had mucocutaneous manifestations. Diplopia, dysphagia and proximal muscle weakness were presenting symptoms of myasthenia in majority, myasthenia crisis was reported in four cases. Positive anti-AChR antibodies were seen in almost all patients. Anticholinesterases were used for all patients. Plasmapheresis was done for one of the cases, who presented with a crisis, like in our case. Two patients relapsed, despite neostigmine necessitating the administration of intravenous immunoglobulin. One patient with myasthenic crisis died after 5 years.

Jallouli et al. reported 17 patients with concomitant SLE and MG. The mean age at MG onset and SLE diagnosis was 34.5 (14–64) and 37.8 (18–72) years, respectively. The presenting symptoms of MG were limb weakness (94%), ocular (88%) and bulbar involvement (53%). Autoantibodies against the acetylcholine receptor were positive in 94% of cases. The main manifestations of SLE included arthritis (88%), cytopenias (53%) and skin rash (41%). Treatment of SLE required hydroxychloroquine (94%), steroids (47%) and immunosuppressive drugs (18%). In this case series, patients with SLE and MG were older at SLE diagnosis, had lower incidence of malar rash, photosensitivity, mucosal ulcers, renal involvement, and central nervous system involvement.^10^ Our patient had predominant arthritis, cytopenia with strong anti-dsDNA antibody positivity and low complement at initial presentation. (**Table 1**)

**Table 1.** Case reports of SLE preceding MG.

**Author**	**Sex**	**Age at SLE onset (years)**	**Age at MG onset (in years)**	**SLE features**	**Myasthenia features**	**Treatment**	**Outcome**
Nagarajan et al.^11^	F	38	38	Arthritis, lupus nephritis, dsDNA positive	Bilateral ptosis, EMG-NMJ defect	Anticholinesterase	Remission
Vaiopoulos et al.^12^	F	18	22	Arthritis Recurrent oral ulcers, ANA positive dsDNA high titres	Proximal muscle weakness Tensilon test positive, Anti-AChR normal	Pyridostigmine	Remission
Barbosa et al.^13^	F	36	36	Oral ulcers, malar rash, and arthritis	Dysphagia, dysphonia, ataxia Anti-AChR positive	Pyridostigmine	Remission
Bhinder et al.^14^	F	28	48	Polyarthritis, rash ANA, Anti dsDNA positive	Ptosis dysphagia Respiratory failure, presented with crisis Anti-AChR, Edrophonium test positive	Pyridostigmine Plasmapheresis Methyl prednisolone	Died after 5 years
Bhinder et al.^14^	F	29	43	Polyarthritis Rash, Seizures, Leukopenia, ANA dsDNA positive	Ptosis, diplopia Anti-AChR positive, Edrophonium test positive	Pyridostigmine	Remission
Casterjon et al.^15^	F	25	41	Fever, arthritis, leukopenia ANA, anti dsDNA positive C_3_/C_4_–decreased, aCL positive	Binocular diplopia, Progressive muscle weakness, dyspnoea, Anti-AChR positive	Pyridostigmine	Remission
Casterjon et al.	F	19	30	Arthritis, serositis, nephritis ANA, anti dsDNA positive	Blurring of vision, ptosis, muscle weakness, Anti-AChR positive	Pyridostigmine	Remission
Present case	F	27	29	Inflammatory arthritis, nephritis, leukopenia ANA, dsDNA positive	Dysphagia, dysphonia, dyspnoea, ocular involvement presented in crisis	Plasma exchange, Pyridostigmine	Remission

Immunological association between Myasthenia gravis and SLE is not clear. Hypothesis includes loss of central tolerance after thymectomy, molecular mimicry and structural similarity between main immunogenic region of α 65–80 of AChR and U1 small nuclear ribonucleoprotein, functional defect of T regulatory cells in thymus, dysregulated expression of FAS (CD95).^16^ CXCL13, a chemokine that activates both B and T lymphocytes, was found in high concentration in both SLE and MG patients compared to control population.^17–19^ Granulocyte-macrophage colony-stimulating factor (GM-CSF), found endogenously as well as exogenously, is a common protective factor between the two autoimmune diseases.^20^ There are conflicting reports on exacerbation of MG after initiation of HCQ in lupus. In the case series by Jaillou et al., eight patients (47%) developed MG after initiation of HCQ. Among them, only one patient showed direct association with HCQ and myasthenia exacerbation, though she was negative for anti-AChR antibody and improved with intravenous immunoglobulins, acetylcholinesterase inhibitors, and withdrawal of HCQ. No firm conclusions could be drawn by authors since acetylcholinesterase inhibitors were not discontinued and HCQ was not re-administered. They also reported that HCQ was well tolerated by most of the patients and the incidence of exacerbation of myasthenia after HCQ treatment in one patient might be due to chance effect. Hydroxychloroquine treatment appears to be safe in this setting. Our patient continued HCQ throughout the myasthenic episode without any deterioration of symptoms.

## CONCLUSION

We report a case of SLE who presented with a postpartum myasthenic crisis. The association between SLE and MG is complex. The presentations may be atypical, and the diagnosis of MG warrants a high index of suspicion. Lupus patients who present with fluctuating muscular weakness specially ptosis and fatigue should be referred for determination of anti-AChR antibodies and neurological examination to exclude MG. Further research is required for better understanding of the association of SLE and MG.

## References

[1] Raut S Reddy I Sahi, Masood A Malik BH. Association Between Systemic Lupus Erythematosus and Myasthenia Gravis: Coincidence or Sequelae? Cureus 2020 Jun 3;12(6):e8422. 32642338 10.7759/cureus.8422 PMC7336596

[2] Nacu A Andersen JB Lisnic V Owe JF Gilhus NE. Complicating autoimmune diseases in myasthenia gravis: a review. Autoimmunity 2015;48(6):362–8. 25915571 10.3109/08916934.2015.1030614 PMC4616023

[3] Mao ZF Yang LX Mo XA Qin C Lai YR He NY Frequency of autoimmune diseases in myasthenia gravis: a systematic review. Int J Neurosci 2011 Mar;121(3):121–9. 21142828 10.3109/00207454.2010.539307

[4] Hrycek A. Systemic lupus erythematosus and myasthenia gravis. Pol Arch Med Wewn 2009;119:582–5. 19776704

[5] Park MJ Kim YA Lee SS Kim BC Kim MK Cho KH. Appearance of systemic lupus erythematosus in patients with myasthenia gravis following thymectomy: two case reports. J Korean Med Sci 2004; 19:134–6. 14966356 10.3346/jkms.2004.19.1.134 PMC2822250

[6] The American College of Rheumatology nomenclature and case definitions for neuropsychiatric lupus syndromes. Arthritis Rheum 1999 Apr;42(4):599–608. 10211873 10.1002/1529-0131(199904)42:4<599::AID-ANR2>3.0.CO;2-F

[7] Sthoeger Z Neiman A Elbirt D Zinger H Magen E Burstein R High prevalence of systemic lupus erythematosus in 78 myasthenia gravis patients: a clinical and serologic study. Am J Med Sci 2006;331(1):4–9. 16415656 10.1097/00000441-200601000-00004

[8] Thorlacius S Aarli JA Riise T Matre R Johnsen HJ. Associated disorders in myasthenia gravis: autoimmune diseases and their relation to thymectomy. Acta Neurol Scand 1989;80(4):290–5. 2816285 10.1111/j.1600-0404.1989.tb03881.x

[9] Bekircan-Kurt CE Tuncer Kurne A Erdem-Ozdamar S Kalyoncu U Karabudak R Tan E. The course of myasthenia gravis with systemic lupus erythematosus. Eur Neurol 2014;72(5–6):326–9. 25323839 10.1159/000365568

[10] Jallouli M Saadoun D Eymard B Leroux G Haroche J Le Thi Huong D The association of systemic lupus erythematosus and myasthenia gravis: a series of 17 cases, with a special focus on hydroxychloroquine use and a review of the literature. J Neurol 2012 Jul;259(7):1290–7. 22160434 10.1007/s00415-011-6335-z

[11] Nagarajan M Maasila AT Dhanapriya J Dineshkumar T Sakthirajan R Rajasekar D Systemic Lupus Erythematosus and Myasthenia Gravis: A Rare Association. Indian J Nephrol 2019 Jan–Feb;29(1):62–4. 30814797 10.4103/ijn.IJN_12_18 PMC6375012

[12] Vaiopoulos G Sfikakis PP Kapsimali V Boki K Panayiotidis P Aessopos A The association of systemic lupus erythematosus and myasthenia gravis. Postgrad Med J 1994;70:741–5. 7831174 10.1136/pgmj.70.828.741 PMC2397764

[13] Barbosa RE Córdova S Cajigas JC. Coexistence of systemic lupus erythematosus and myasthenia gravis. Lupus 2000;9(2):156–7. 10787017 10.1191/096120300678828172

[14] Bhinder S Majithia V Harisdangkul V. Myasthenia gravis and systemic lupus erythematosus: truly associated or coincidental-two case reports and review of the literature. Clin Rheumatol 2006 Jul;25(4):555–6. 16328087 10.1007/s10067-005-0099-8

[15] Castrejón I Shum K Tseng CE Askanase A. Association between myasthaenia gravis and systemic lupus erythematosus: three case reports and review of the literature. Scand J Rheumatol 2011 Nov;40(6):486–90. 21722072 10.3109/03009742.2011.575077

[16] Moulian N Bidault J Truffault F Yamamoto AM Levasseur P Berrih-Aknin S. Thymocyte Fas expression is dysregulated in myasthenia gravis patients with anti-acetylcholine receptor antibody. Blood 1997;89(9):3287–95. 9129034

[17] Lee HT Shiao YM Wu TH Chen WS Hsu YH Tsai SF Serum BLC/CXCL13 concentrations and renal expression of CXCL13/CXCR5 in patients with systemic lupus erythematosus and lupus nephritis. J Rheumatol 2010 Jan;37(1):45–52. 19955043 10.3899/jrheum.090450

[18] Shiao YM Lee CC Hsu YH Huang SF Lin CY Li LH Ectopic and high CXCL13 chemokine expression in myasthenia gravis with thymic lymphoid hyperplasia. J Neuroimmunol 2010 Apr 15;221(1–2):101–6. 20223524 10.1016/j.jneuroim.2010.02.013

[19] Meraouna A Cizeron-Clairac G Panse RL Bismuth J Truffault F Tallaksen C The chemokine CXCL13 is a key molecule in autoimmune myasthenia gravis. Blood 2006;108:432–40. 16543475 10.1182/blood-2005-06-2383 PMC1847364

[20] Sheng JR Li L Ganesh BB Vasu C Prabhakar BS Meriggioli MN. Suppression of experimental autoimmune myasthenia gravis by granulocyte-macrophage colony-stimulating factor is associated with an expansion of FoxP3+ regulatory T cells. J Immunol 2006;177:5296–306. 17015715 10.4049/jimmunol.177.8.5296

